# Classification and Taxonomy of Vegetable Macergens

**DOI:** 10.3389/fmicb.2015.01361

**Published:** 2015-11-27

**Authors:** Bukola R. Aremu, Olubukola O. Babalola

**Affiliations:** ^1^Department of Biological Sciences, Faculty of Agriculture, Science and Technology, North-West University, Mmabatho, South Africa; ^2^Food Security and Safety Niche Area, Faculty of Agriculture, Science and Technology, North-West University, Mmabatho, South Africa

**Keywords:** classification, macergens, pectolytic, proteolytic, taxonomy, species

## Abstract

Macergens are bacteria capable of releasing pectic enzymes (pectolytic bacteria). These enzymatic actions result in the separation of plant tissues leading to total plant destruction. This can be attributed to soft rot diseases in vegetables. These macergens primarily belong to the genus *Erwinia* and to a range of opportunistic pathogens namely: the *Xanthomonas* spp., *Pseudomonas* spp., *Clostridium* spp., *Cytophaga* spp., and *Bacillus* spp. They consist of taxa that displayed considerable heterogeneity and intermingled with members of other genera belonging to the *Enterobacteriaceae*. They have been classified based on phenotypic, chemotaxonomic and genotypic which obviously not necessary in the taxonomy of all bacterial genera for defining bacterial species and describing new ones These taxonomic markers have been used traditionally as a simple technique for identification of bacterial isolates. The most important fields of taxonomy are supposed to be based on clear, reliable and worldwide applicable criteria. Hence, this review clarifies the taxonomy of the macergens to the species level and revealed that their taxonomy is beyond complete. For discovery of additional species, further research with the use modern molecular methods like phylogenomics need to be done. This can precisely define classification of macergens resulting in occasional, but significant changes in previous taxonomic schemes of these macergens.

## Introduction

Marcergens are soft rot causing bacteria, responsible for plant tissue maceration resulting in total tissue collapse (Beattie, 2006; Bhai et al., 2012). Soft rot diseases of vegetables are the most characteristic symptom of tissue maceration in a plant. These begin as small water soaked lesion, expands and intensifies until the tissue turns soft and watery (Reddy, 2015). Apparently, the outer surface of the diseased plant might stay unbroken, while tanning and depressed, or enclosed in an exuding bacterial mucus layer (Heyman et al., 2013). Foul smells are common owing to the discharge of explosive complexes through tissue degradation. Best bacterial growth follows plant cell lysis in these diseases (Rich, 2013). Soft-rotting bacteria are distinguished for the speed at which they stimulate soft rot. Warehoused crop may turn to liquid in only a few hours (Reddy, 2015). These pathogens usually enter through wound spots or natural openings such as lenticels and persist in the intercellular spaces and vascular tissues till the environmental conditions become fit for disease development. Parenchymatous tissues are macerated by massive quantities of pectic exoenzymes exudates produced during this period. These enzymes comprise of cellulolytic enzymes, pectate lyases, and pectin methylesterases, which are responsible for the total tissue destruction (Parthiban et al., 2012).

**FIGURE 1:**
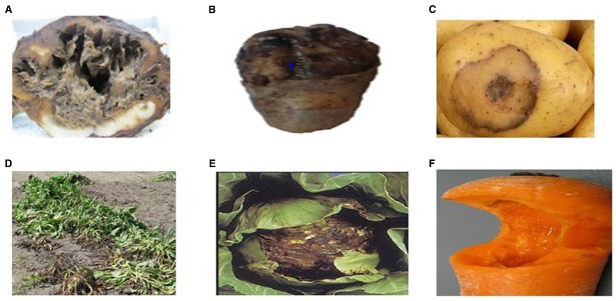
**Unmarketable Vegetable as a Result of Macergen Infestation (A).** Chicory root affected by soft rot diseases, **(B,C)**. Potato with soft rot diseases, **(D)**. Chicory leaves with soft rot disease, **(E)**. Cabbage with soft rot disease, **(F)**. Carrot with soft rot disease. Adapted from Lindsey du Toit, Washington State University, North Carolina Cooperative Extension Sevice (Lan et al., 2013).

Soft rot can be found worldwide, anywhere ample storage tissues of vegetables and ornamentals are found (Golkhandan et al., 2013; Elbanna et al., 2014). Potatoes, carrots, and onions are among the most affected vegetables, along with tomato and cucumber (Mir et al., 2010) (Figure 1). Soft rot of fleshy vegetables and ornamental plants can be caused by more than six genera of pectolytic bacteria comprising; *Erwinia*, *Pseudomonas*, *Clostridium*, *Bacillus*, *Cytophaga*, and *Xanthomonas* (Elbanna et al., 2014). The estimated rate of infection of macergens on harvested crop ranges from 15 to 30%. *Erwinia* are the major macergens causing tissue degradation in vegetables (Choi and Kim, 2013; Waleron et al., 2014). Although, *Erwinia* are the primary macergens, it is not a single taxon. It is reclassified into genera such as *Pectobacterium* and *Dickeya* (Brady et al., 2012; Nabhan et al., 2012; Czajkowski et al., 2013). Macergens comprise of multiple groups ranging from the very complex *Pseudomonas*, a gamma-*proteobacteria* to as diverse as *Bacillus* and *Clostridium* which are firmicutes. *Bacillus* spp., *Clostridium* spp., *Pseudomonas marginalis*, and *Pantoea agglomerans* only cause soft rot when conditions are favorable to do so, thus are secondary invader called opportunistic pathogens (da Silva, 2013). Among all these pectolytic bacteria, soft rot *Erwinias* are the most important primary macergens that can macerate both the growing and harvested crop (Baz et al., 2012). All other bacteria are referred to as secondary because they can only destroy the parenchymatous tissues of plant under extreme environmental conditions or secondary invaders after *Erwinias* or other pathogens have infected the plant.

These macergens infect and destroy plant tissues both pre- and post-harvest and this species causes the greatest damage to harvested vegetables (Lee et al., 2012). There is need to ensure a continuous cold chain from immediately after post-harvest, to retail for successful management of these ubiquitous spoilage bacteria that only thrive well at temperatures of 20°C and above (Tournas, 2005). The fluorescent *Pseudomonads* (*P. fluorescens* and *P. viridiflava*) can macerate plant parenchymatous tissues at a temperature below 4°C. This cause higher occurrence of these bacteria on decayed vegetables both at wholesale and retail markets. These soft-rotting fluorescent *Pseudomonads* and *Erwinia* therefore become the major threat to commercial fresh product operations and fresh vegetables precisely, from the farm to retail and wholesale outlets (Saranraj et al., 2012). There are currently no commercial agents available specifically for controlling soft rot (Yaganza et al., 2014).

Despite advances in vegetable production and disease management, many challenges face growers of vegetables, out of which the major one is the damage caused by macergens (Wu et al., 2012). Macergens damage the tissues of vegetable thereby reducing the quality, yield, shelf-life and consumer satisfaction of these plants (Akhtar, 2015). They usually cause great economic losses due to their ability to infect and macerate vegetable tissues at any point in time, be it, the field, transit, storage or marketing period (Lee et al., 2012). In the nature of today’s worldwide market, there are extremely high expectations for growers to provide ample supplies of high-quality, disease-free produce that have extended shelf-life (Kewa, 2012; Cheverton, 2015). The traditional methods to identify these macergens are extremely slow, more complex and obsolete (Hawks, 2005). Also, resistance genes active against macergens have been found in multiple host species, but their sequences and mechanisms remain unknown (Nykyri et al., 2012). Hence, means of quick identification of these bacteria are very essential. But the understanding of the taxonomy of these macergens will go a long way in shedding light to understand their biology and ultimately to the best method of controlling them. At present, there is very few knowledge available on the biology, ecology and epidemiology of macergens affecting vegetables in lowland and highland tropics. In order to increase crop production an assessment of biology, ecology and epidemiology of these bacteria need to be successfully implemented. Thus, this review focuses on the classification and taxonomy of the macergens to the species level. This is very important for more exploration in biotechnology.

## Types of Microorganisms on Vegetables

The majority of Gram negative rods identified from raw vegetables were fluorescent *Pseudomonads* spp., *Klebsiella* spp., *Serratia* spp., *Flavobacterium* spp., *Xanthomonads* spp., *Chronobacterium* spp., and *Alcaligenes* (Elbanna et al., 2014). In vegetables like broccoli, cabbage, mungbean sprouts and carrot, Gram positive rods were predominantly isolated. *Coryneform* bacteria and catalase negative *cocci* were also predominantly isolated from broccoli, raw peas and raw sweet corn. In India, the mesophilic microflora of potatoes mainly comprised Gram positive bacteria, *Bacillus* spp., and *Micrococcus* spp. as fluorescent *Pseudomonads*, *Cytophaga* spp., *Flavobacterium* spp., *Xanthomonas*. spp., and *Erwinia* spp. *Leuconostoc mesenteroides* was the most common and abundant species found in vegetables amongst lactic acid bacteria (Andrews and Harris, 2000).

## Taxonomy of Macergens

### Genus *Erwinia*

*Erwinia* belongs to the phylum *Proteobacteria*, class *Gammaproteobacteria*, order *Enterobacteriales* and family *Enterobacteriaceae*. For the past several decades, *Enterobacteria* that macerate and decay plants tissues, often referred to as the pectolytic Erwinias, were placed in genus *Erwinia*. Named after the eminent plant pathologist, Erwinin F. Smith. They are non-spore forming, facultative Gram negative rod-shaped anaerobes of approximately 0.7 × 1.5 μm in size with peritrichous flagella. This genus contains diverse set of group of organisms represented in Table 1. Since its establishment many new genera have been generated from *Erwinia*.

**TABLE 1:** **List of interesting *Erwinia* species**.

***Erwinia* species**	**Sources**	**Reference**
*Erwinia amylovora*	Apple, pear	Ashmawy et al. (2015)
*Erwinia ananas*	Honeydew melon	Wells et al. (1987)
*Erwinia cacticida*	Sunflower	Valenzuela-Soto et al. (2015)
*Erwinia carotovora*	Carrots, potatoes, cucumbers, onions, tomatoes, lettuce	Nazerian et al. (2013), Akbar et al. (2015)
*Erwinia chrysanthemi*	Potatoes	van der Wolf et al. (2014)
*Erwinia papaya*	Papaya	Gardan et al. (2004)
*Erwinia cypripedii*	Papaya	Leu et al. (1980)
*Erwinia herbicola*	Tomatoes	Ibrahim and AL- Saleh (2010)
*Erwinia mallotivora*	Papaya	Amin et al. (2011)
*Erwinia nigrifluens*	Walnut, hazelnut	Frutos (2010)
*Erwinia persicinus*	Bananas, cucumbers, and tomatoes	O’Hara et al. (1998)
*Erwinia psidii*	Guava, Eucalyptus	Pomini et al. (2005), Coutinho et al. (2011)
*Erwinia quercina*	Oaks	Shang et al. (2015)
*Erwinia rhapontici*	Rhubarb, garlic, tomato, onions, cucumber	Dowson (1941), Huang et al. (2003)
*Erwinia rubrifaciens*	Walnut, hazelnut	Frutos (2010)
*Erwinia stewartii*	Sweet corn	Roper (2011)
*Erwinia tracheiphila*	Pumpkin, watermelon	Sanogo et al. (2011)
*Erwinia uredovora*	Rice	Yan et al. (2010)
*Erwinia tasmiensis*	Pear	Thapa et al. (2012)
*Erwinia billingiae*	Pear	Kube et al. (2005)
*Erwinia wasabiae*	Potatoes	Moleleki et al. (2013)
*Erwinia brasiliense*	Potatoes	van der Merwe et al. (2010)
*Erwinia betavasculorum*	Sugarbeet	Nedaienia and Fassihiani (2011)
*Erwinia oleae*	Olive	Moretti et al. (2011)
*Erwinia pyrifoliae*	Pear	Shrestha et al. (2003)
*Erwinia atrosepticum*	Potatoes	Kwasiborski et al. (2013)
*Erwinia uzenensis*	Pear	Matsuura et al. (2012)
*Erwinia odoriferum*	Chicory, potato	Waleron et al. (2014)
*Erwinia piriflorinigrans*	Pear	López et al. (2011)
*Erwinia toletana*	Olive	Rojas et al. (2004)

#### Nomenclature of *Erwinia*

Traditionally two species (*Erwinia carotovora* and *Erwinia chrysanthemi*) are circumscribed as the important plant pathogenic strains, but has been reclassification into a new genus, *Pectobacterium*, with multiple species being proposed (Gardan et al., 2003). *Pectobacterium* spp. (Waldee, 1945; formerly *Erwinia carotovora*) and *Dickeya* spp. (formerly *Erwinia chrysanthemi*) species are related to soft rot *Enterobacteria* pathogens with broad host ranges. These species formerly were known as the soft rot *Erwinia* spp., but several studies have shown that the soft rot *Enterobacteria* and *E. amylovora*, the type strain of the *Erwinia* genus, are too divergent to be included in one clade. Therefore, the soft rot *Erwinia* spp. were moved to two new genera as *Pectobacterium* and *Dickeya* (Nabhan et al., 2013). *Pectobacterium* and *Dickeya* spp. are considered broad-host range pathogens in part because, they have been isolated from so many plant species and in part because, single strains are pathogens of numerous plant species under experimental conditions. Within the genus *Pectobacterium*, there are five major clades designated I, II, III, IV, and V, which differs from previous studies. These comprise five subspecies or species-level clades of *Pectobacterium* namely; *Pectobacterium carotovorum* subsp. *carotovorum* (syn. *Erwinia carotovorum* subsp. *carotovorum*), *Pectobacterium atrosepticum* (syn. *Erwinia carotovorum* subsp. *atrosepticum*) *Pectobacterium wasabiae* (syn. *Erwinia carotovorum* subsp. *wasabiae*), *Pectobacterium betavasculorum* (syn. *Erwinia carotovorum* subsp. *betavasculorum*), and *Pectobacterium carotovorum* subsp. *brasiliense* (Hauben et al., 2005; Nabhan et al., 2012). The reconstructed phylogenies agree that *P. atrosepticum*, *P. betavasculorum*, and *P. wasabiae* do form individual clades and place the *brasiliensis* strains in an individual clade.

Previous suggestions to separate the pectolytic *Enterobacteria* into the genus *Pectobacterium* has not found favor among phytobacteriologists. Initially the suggestion was made by Waldee (1945), who recommended the segregation on the basis of the unique pectolytic activity of the bacteria. Consequently, Hauben et al. (1998) revived the suggestion to support the proposal by adding evidence from the 16S ribosomal DNA sequence analysis of various plant-associated members of the *Enterobacteriaceae*. Although the phenotypic characterization and analysis of a single DNA fragment might have been considered insufficient for the subdivision at the generic level, the DNA-DNA hybridization study conducted by Gardan et al. (2003) provides further stimulation to change in favor of the new nomenclature. Samson et al. (2005), have proposed several new species from new genus, *Dickeya* for *E. chrysanthemi*, comprising of six genomic species namely: *Dickeya dianthicola*, *D. dadantii*, *D. zeae*, *D. chrysanthemi*, *D. dieffenbachiae*, *D. paradisiaca*.

A recently initiated multi-locus sequencing project, as well as DNA hybridization data from the 1970s, supports the transfer of *E. carotovora* and *E. chrysanthemi* to two separate genera as well as the elevation of some soft rot *Erwinia* subgroups to the species level (Brady et al., 2012).

All the phylogenetic analyses completed to date have suffered from the small number of strains available for some *Enterobacteria* species, which makes it difficult to determine the relatedness of these taxa. Unfortunately, the naming and re-naming of species has caused considerable confusion in the literature, resulting in manuscripts being published with names that were used for only a few years. Since *Erwinia* has remained the preferred name used in the literature, the comprehensive phylogenetic study of the entire group of soft rot *Enterobacteria* remains uncompleted (Charkowski, 2006; Elbanna et al., 2014). The pectolytic *Erwinia* are ubiquitous in environments that support plant growth, and because they may be found in association with asymptomatic plants, they have been viewed as opportunistic pathogens analogous to medical bacteria that infect only immunologically compromised individuals. *Pectobacterium carotovorum*, in the family *Enterobacteriaceae*, is a highly diverse species consisting of at least two valid names, *P. carotovorum* subsp. *carotovorum* and *P. carotovorum* subsp. *odoriferum* and a suggested third taxon, *P*. *carotovorum* subsp. *brasiliense* (De Boer et al., 2012). Despite the lack of valid *carotovorum* publication, the *P. carotovorum* subsp. *brasiliense* name has been used in more than 10 publications since first published in 2004 as *Erwinia carotovora* subsp. *brasiliense* (Ma et al., 2007). Assigning strains to this taxon was based mainly on the genetic information of the 16S-23S intergenic spacer region of the rRNA operon, partial sequence of 16S rRNA gene and multilocus sequence analysis (MLSA) of housekeeping genes and MALDI-TOF characterization (Wensing et al., 2012). Table 2 depicts the molecular method employed in the characterization of *Pectobacterium* and *Dickeya* species. *Pectobacterium carotovorum* subsp. *brasiliense* was first described as causing blackleg disease on potatoes (*Solanum tuberosum* L.) in Brazil and has since been described as also causing soft rot in *Capsicum annum* L., *Ornithogalum* spp., and *Daucus carota* subsp. *Sativus*. Strains of this taxon were isolated in the USA, Canada, South Africa, Peru, Germany, Japan, Israel, and Syria (Ngadze et al., 2012; Moleleki et al., 2013).

**TABLE 2:** **Molecular methods of identifying macergens**.

**Macergens**	**Molecular methods**	**Isolation sources**	**Reference**
*Pectobacterium carotovora*	AFLP, MLSA, MLST, PFGE, MALDI-TOF MS, qPCR	Potatoes	Nabhan et al. (2012), Ngadze et al. (2012), Šalplachta et al. (2015), Humphris et al. (2015)
*Pectobacterium atrosepticum*	AFLP, RFLP, RAPD, qPCR, MALDI-TOF MS	Potatoes	Ngadze et al. (2012), Duarte et al. (2004), Pritchard et al. (2013), Šalplachta et al. (2015)
*Pectobacterium wasabiae*	AFLP, MLST, RAPD, qPCR	Horse radish, potatoes, crucifer	Avrova et al. (2002), De Boer et al. (2012), Kim et al. (2012)
*Pectobacterium odoriferum*	AFLP, MLSA, MLST	Potatoes, celery	Avrova et al. (2002), Waleron et al. (2014)
*Pectobacterium betavasculorum*	AFLP, MLST, 16S rRNA, qPCR	Potatoes	Avrova et al. (2002), De Boer et al. (2012), van der Merwe et al. (2010), Humphris et al. (2015)
*Pectobacterium brasiliense*	MLST, 16S-23S rDNA, qPCR, MALDI-TOF MS	Potatoes	De Boer et al. (2012), Czajkowski et al. (2015), Werra et al. (2015)
*Dickeya chrysanthemi*	16S—23S rDNA, RFLP of recA, AFLP, rep-PCR, 16S rDNA, MLST, DNA–DNA hybridization, qPCR, MALDI-TOF MS	Potatoes	Laurila et al. (2008), Waleron et al. (2002), Avrova et al. (2002), Słwiak et al. (2009), Ma et al. (2007), Samson et al. (2005), Pritchard et al. (2013), Šalplachta et al. (2015)
*Dickeya dianthicola*	rep-PCR, 16S rDNA, PFGE, MALDI-TOF MS, DNA–DNA hybridization, qPCR,	Potatoes	Słwiak et al. (2009), Degefu et al. (2013), Šalplachta et al. (2015), Samson et al. (2005), Pritchard et al. (2013)
*Dickeya dadantii*	rep-PCR, 16S rDNA, PFGE, DNA–DNA hybridization, qPCR, MALDI-TOF MS	Potatoes,	Słwiak et al. (2009), Degefu et al. (2013), Samson et al. (2005), Pritchard et al. (2013), Šalplachta et al. (2015)
*Dickeya zeae*	rep-PCR, 16S rDNA, RPLP, PFGE, DNA–DNA hybridization, qPCR, MALDI-TOF MS	Potatoes, maize	Słwiak et al. (2009), Samson et al. (2005), Degefu et al. (2013), Pritchard et al. (2013), Šalplachta et al. (2015)
*Dickeya dieffenbachiae*	rep-PCR, 16S rDNA, AFLP, PFGE, DNA–DNA hybridization, MALDI-TOF MS	Potatoes	Słwiak et al. (2009), Samson et al. (2005), Degefu et al. (2013), Šalplachta et al. (2015)
*Dickeya paradisiaca*	rep-PCR, 16S rDNA, AFLP, PFGE, qPCR, MALDI-TOF MS	Potatoes, banana, maize	Słwiak et al. (2009), Degefu et al. (2013), Samson et al. (2005), Pritchard et al. (2013), Šalplachta et al. (2015)
*Dickeya solani*	rep-PCR, PFGE, RFLP, qPCR, MALDI-TOF	Potatoes, tomato, maize,	van der Wolf et al. (2014), Degefu et al. (2013), Waleron et al. (2013a), Pritchard et al. (2013), Šalplachta et al. (2015)

*PFGE: Pulse-field gel electrophoresis; 16S-23S intergenic transcribed region of the rRNA operon; MLSA: multilocus sequence analysis of housekeeping genes; MALDI-TOF MS: matrix-assisted laser desorption/ionization time-of-flight mass spectrometry; AFLP: amplified fragment length polymorphism; MLST: multilocus sequence tagging; RFLP: restriction fragment length polymorphism; RAPD: random amplification of polymorphic DNA; rep-PCR: repetitive sequence-based PCR 3.2 Genus Pseudomonas*.

Genus *Pseudomonas* was first described in 1894 as one of the most diverse and ubiquitous bacterial genera whose species have been isolated worldwide from soil, decayed plant materials and rhizopheric region, quite a numerous plant species (Migula, 1894). They comprise a heterogeneous group of species which were grouped into five groups based on RNA homology (Saranraj et al., 2012). The RNA-homology group I belong to the fluorescent group because of their ability of producing pyoverdines. Pectolytic *Pseudomonas* belongs to this rRNA group I organism of gamma *Proteobacteria*. They are non-sporulating, Gram-negative, strict aerobic, rod-shape with polar flagella (Özen and Ussery, 2012). The strains of these bacteria called *P. marginalis* or *P. fluorescens* can be attributed to soft rot diseases in vegetables. The very complex groups of fluorescent, oxidase positive soft rot *Pseudomonas* are opportunistic macergens. Table 3 represents the molecular methods for the description of *Pseudomonas* species belonging to macergens.

**TABLE 3:** **Molecular methods for the description of *Pseudomonas* species belonging to macergens**.

**Macergens**	**Molecular methods**	**Isolation sources**	**Reference**
*Pseudomonas. fluorescens*	RFLP ITS1, 16S rRNA gene, WC-MALDI-TOF MS	Wheat	Franzetti and Scarpellini (2007), Mulet et al. (2012)
*Pseudomonas marginalis*	16S rRNA	Onion	Achbani et al. (2014)
*Pseudomonas putida*	16S rRNA, MLSA	Potato	Delfan et al. (2012), Mulet et al. (2010)
*Pseudomonas chlororaphis*	16S rRNA, MLSA WC-MALDI-TOF MS	Sugarbeet and spring wheat	Mulet et al. (2010), Mulet et al. (2012)
*Pseudomonas aureofaciens*	16S rRNA, MLSA, WC-MALDI-TOF MS	Corn	Mulet et al. (2010), Mulet et al. (2012)
*Pseudomonas syringae*	16S–23S rDNA, 16S rRNA, MLSA	Kiwifruit, cucumber, tomato	Rees-George et al. (2010), Mulet et al. (2010)
*Pseudomonas stutzeri*	16S rRNA, MLSA	Ginseng	Mulet et al. (2010)
*Pseudomonas aeruginosa*	RFLP ITS1, 16S rRNA gene, MLST	Tomato, lettuce, celery	Franzetti and Scarpellini (2007)
*Pseudomonas pertucinogena*	16S rRNA, MLSA	Wheat	Mulet et al. (2010)
*Pseudomonas aurantiaca*	16S rRNA, MLSA, WC-MALDI-TOF MS	Cotton	Mulet et al. (2010), Mulet et al. (2012)
*Pseudomonas corrugata*	rep-PCR fingerprinting, MLSA	Tomato	Trantas et al. (2015)
*Pseudomonas cichorii*	16S rRNA, MLSA	Tomato	Mulet et al. (2010)

*16S-23S intergenic transcribed region of the rRNA operon; MLSA: multilocus sequence analysis of housekeeping genes; MALDI-TOF MS: matrix-assisted laser desorption/ionization time-of-flight mass spectrometry; AFLP: amplified fragment length polymorphism; MLST: multilocus sequence tagging; RFLP: restriction fragment length polymorphism; rep-PCR: repetitive sequence-based PCR*.

#### Nomenclature of *Pseudomonas*

The nomenclature of bacteria in the genus *Pseudomonas* has changed considerably during the last decennia. *P. marginalis* or *P. fluorescens* are pectinolytic that cause strains soft rot on a wide range of hosts. The taxonomic and phytopathogenic status of *P. marginalis* is not well known. However, these are biochemically and phenotypically indistinguishable from saprophytic strains of *P. fluorescens* biovars II, *P. putida*, and *P. chlororaphis* (now includes *P. aureofaciens*). Based on their ability to degrade pectin and macerate the plant parenchymatous tissues they are referred to as *P. marginalis*. Recently, based on 16S rRNA analysis Anzai et al. (2000) came up with 57 strains of *Pseudomonas* sensu stricto with seven subclusters: *P. syringae* group, *P. chlororaphis* group, *P. fluorescens* group, *P. putida* group, *P. stutzeri* group, *P. aeruginosa* group, and *P. pertucinogena* group (Novik et al., 2015). Also, in the same genus *Pseudomonas*, some species have been found to be misclassified for instance *P. aureofaciens* and *P. aurantiaca*, which were reclassified into *P. chlororaphis* (Peix et al., 2007).

Ever since the discovery of genus *Pseudomonas*, it has undergone several taxonomic changes not only as far as the number of species included, but also as far as the criteria used for their definition and delineation. In Bergey’s Manual of Systematic Bacteriology’s current edition, an extensive list of methods used in *Pseudomonas* taxonomy was integrated (Palleroni, 2005). These methods, which consist of cell morphology and structure, cell wall composition, pigment types, nutritional and metabolic characteristics, susceptibility to different compounds, antibiotic production, pathogenicity of other organisms, antigenic structure and genetic and ecological studies revealed the efforts for characterizing *Pseudomonas* species. The phenotypic taxonomic markers comprise a set of tests, namely: cell shape, flagella type, consumption of carbon sources such as organic acids, polyalcohols and amino acids, ability to grow in different culture conditions, antibiotic resistance, production of antibiotic substances and exocellular enzymes (Palleroni, 2005).

In *Pseudomonas* taxonomy, the effectiveness of chemotaxonomic studies has been confirmed, such as quinone systems, fatty acid, protein, polar lipid or polyamine profiles, which are usually useful in the taxonomy of most bacterial groups. Generally, *Pseudomonas* species were reclassified by chemotaxonomic markers into other genera such as *P. mephitica* into *Janthinobacterium lividum* (Kämpfer et al., 2008). Janse et al. (1992), used whole fatty acid analysis in the study of a broad collection opportunistic phytopathogenic to clarify the taxonomic position of some *P. marginalis* strains included in the *P. fluorescens* group. Also, Janse et al. (1992) reported that other bacteria (*P. putida*, *P. aureofaciens*, and *P. tolaasii*) within the fluorescent oxidase positive pseudomonads group also exhibit pectinolytic ability. Hence, they are referred to as *P. fluorescens* supercluster. The study of polyamine composition in Proteobacteria revealed putrescine as the main polyamine present in the *P. fluorescens* complex, thus help in the delineation of species from this group. Recently, the polar lipid patterns of representative species of genus *Pseudomonas* were analyzed which showed the presence of phosphatidylglycerol, diphosphatidylglycerol and phosphatidylethanolamine as major polar lipids (Cámara et al., 2007).

Siderotyping an interesting taxonomic tool was used in characterizing fluorescent and then to non-fluorescent based on the isoelectrophoretic. Characterization of the major siderophores and pyoverdines and determination of strains pyoverdine mediated iron uptake specificity led to characterization of several *Pseudomonas* strains at species level, through species-specific pyoverdines (Novik et al., 2015). Mass spectrometry for the determination of molecular mass of pyoverdines has helped recently to improve siderotyping resolution power and accuracy (Meyer et al., 2008).

Currently fluorescent spectroscopy fingerprinting, the most modern techniques for biomolecules analysis are being applied to *Pseudomonas* taxonomy, by emission spectra of three intrinsic fluorophores (NADH, tryptophan, and the complex of aromatic amino acids and nucleic acid), which have been able to differentiate *Pseudomonas* at genus level from *Burkholderia*, *Xanthomonas*, or *Stenotrophomonas* with very high sensitivity, and moreover at species level *P. chlororaphis*, *P. lundensis*, *P. fragi*, *P. taetrolens* and *P. stutzeri* grouped separately from *P. putida*, *P. pseudoalcaligenes*, and *P. fluorescens*, which correlate with the phylogenetic clusters earlier obtained by Anzai et al. (2000); Peix et al. (2007), and Tourkya et al. (2009).

Hence, other gene sequences like housekeeping genes have been used in the last decade as phylogenetic molecular markers in taxonomic studies such as the *rec*A, *atp*D, *car*A, *gyr*B, and *rpo*B, whose effectiveness has been demonstrated in genus *Pseudomonas* for species differentiation (Hilario et al., 2004). For instance, the effectiveness of *rpo*B has been reported in discriminating closely related *Pseudomonas*, with a phylogenetic resolution of the *rpo*B tree roughly three times higher than that of the 16S rRNA gene tree (Tayeb et al., 2005). These genes also enhanced differentiation of subspecies within *P. chlororaphis* (Hilario et al., 2004; Peix et al., 2007). Nevertheless, the analysis of housekeeping genes is not frequently used so far in *Pseudomonas* species description, but only *gyr*B, *rpo*B and *rpoD* have been integrated in the current description of *P. xiamenensis* (Lai and Shao, 2008).

16S-23S rRNA intergenic spacer is another phylogenetic marker used increasingly in taxonomic studies for discrimination of very closely related bacteria, at species and intraspecific levels, even at the strain level because of its high variability both in size and sequence (Sakamoto et al., 2001). This region can be amplified by using universal primers, and specific protocols (Locatelli et al., 2002). The efficacy of this phylogenetic marker has been reported in the differentiation of *Pseudomonas* species (Guasp et al., 2000). The selection of the minimal principles necessary for species delineation and description is selected for each bacterial genus by a committee created by experts in the given genus. The methods used in the taxonomy of the genus *Pseudomonas* and its related genera have been standardized by the subcommittee on the taxonomy. However, the minimal standards for genus *Pseudomonas* species description are yet to be cleared after the 2002 meeting of this subcommittee (De Vos and Yabuuchi, 2002). Hence, the new species description of this genus must be based on the general minimal standards for bacterial species characterization (Stackebrandt et al., 2002). These general minimal standards needed for the classification of new species and/or subspecies must comprise 16S rRNA sequencing, DNA-DNA hybridization, fatty acid analysis and phenotypic classification.

### Genus *Xanthomonas*

The genus *Xanthomonas* belong to the family *Xanthomonadaceae*. This family composed of 10 genera that dwell in an extreme environment. The genus *Xanthomonas* belongs to the gamma proteolytic subdivision (Mbega et al., 2014). They are Gram-negative, aerobic, rod-shape, motile, non-spore forming with a single polar flagellum, comprises of 27 species infecting more than 400 dicots and monocots plant species (Rodriguez et al., 2012).

#### Nomenclature of *Xanthomonas*

Traditionally, genus *Xanthomonas* is referred to as a taxon of pathogenic plant bacteria (Dye et al., 1974; Bradbury, 1984). *Xanthomonas* usually produce some extracellular polysaccharide namely: xanthan and xanthomonadin, a membrane-bound, brominated, aryl-polyene, yellow pigment (Adriko et al., 2014). This yellow pigment is responsible for their pathogenicity and virulence (Subramoni et al., 2006). However, the yellow-pigmented *X.* spp. (*X. campestris*) are the only one associated with tissue maceration of the post-harvest vegetables and fruits (Liao and Wells, 1987). They are opportunistic macergens because they are entering through natural openings or after infection of the plant by *Erwinia* spp.

Genetically, it can be differentiated into over 141 pathovars (pv.) based on specificity range (Swings and Civerolo, 1993). But *Xanthomonas* classification of *X*. *campestris* pathovar was based on the host pathogenicity system (Table 4)

**TABLE 4:** **Macergens host pathogenicity**.

**Macergens**	**Disease symptoms**	**Host range**	**Reference**
*Erwinia carotovora*	Soft rot	Wide	Nabhan et al. (2012), Nabhan et al. (2013)
*Erwinia carotovora ssp. atrosepticum*	Soft rot	Potato	Baz et al. (2012), Ngadze et al. (2012)
*Erwinia carotovora ssp. brasiliensis*	Soft rot	Potato	Moleleki et al. (2013), Zhao et al. (2013)
*Erwinia carotovora ssp. carotovora*	Soft rot	Sugar beet	Waleron et al. (2013b)
*Erwinia carotovora ssp. odorifera*	Soft rot	Chicory	Lan et al. (2013)
*Erwinia carotovora E. chrysanthemi*	Soft rot	Wide	Brady et al. (2012)
*Erwinia cypripedii*	Brown rot	Cypripedium	Horst (2013)
*Erwinia rhapontici*	Crown rot	Rhubarb	Brady et al. (2012)
*Erwinia carcinogenesis*	Soft rot	Giant cactus	Ma et al. (2007)
*Pseudomonas marginalis*	Soft rot	Lettuce, cabbage	Gašic et al. (2014)
*Pseudomonas fluorescens*	Soft rot	Pepper, potato	Bhai et al. (2012), Czajkowski et al. (2012)
*Pseudomonas viridiflava*	Soft rot	Carrot, pepper,	Almeida et al. (2013), Mitrev et al. (2014)
*Pseudomonas putida*	Soft rot	Lettuce, ginger	Krejzar et al. (2008), Moreira et al. (2013)
*Xanthomonas campestris*	Black rot	Crucifers	Kifuji et al. (2013), Vicente and Holub (2013)
*Xanthomonas campestris*	Soft rot	Tomato, pepper	Singh et al. (2012)
*Xanthomonas. campestris aberrans*	Soft rot	Brassica	Gupta et al. (2013)
*Xanthomonas axonopodis vesicatoria*	Soft rot	Tomato	Sharma and Agrawal (2014)
*Xanthomonas axonopodis phaseoli*	Black rot	Bean	Porch et al. (2012), Dutta et al. (2013)
*Xanthomonas axonopodis dieffenbachia*	Soft rot	Tomato, pea	Ismail et al. (2012), Czajkowski et al. (2014)
*Xanthomonas. axonopodis citri*	Soft rot	Potato	Terta et al. (2012)

Initially, this genus undergone diverse taxonomic and phylogenetic studies based on their phenotype and host specificity. Until Vauterin et al. (1995) revised the reclassification of *Xanthomonas* by DNA-DNA hybridization into 20 species based on their genomic relatedness. Phenotypic fingerprinting techniques such as 50S-polyacrylamide gel electrophoresis (50S-PAGE) of cellular proteins and gas chromatographic analysis of fatty acid methyl esters (FAME) reasonable supported these genomic groups to an extent. Hence, both techniques are useful tools in specific and interspecific differentiation of *Xanthomonas* levels (Rademaker et al., 2000).

Other analyses like Multi-Locus Sequence Analysis (MLSA), Amplified Fragment Length Polymorphism (AFLP) were also used in characterisation of this genus, revealing the complexity and diversity of the genus previously described by DNA-DNA hybridization (Ferreira-Tonin et al., 2012; Hamza et al., 2012). Not quite long, the phylogeny of species representing the principal lineages of the genus *Xanthomonas* were reported based on their genome (Rodriguez et al., 2012). The 16S ribosomal DNA sequences and MLSA classified *Xanthomonas* species into two major groups (Vicente and Holub, 2013). Group I comprising: *X. albilineans*, *X. hyacinth*, *X. theicola*, *X. sacchari* and *X. translucens*, and Group II made up of *X. arboricola*, *X. axonopodis*, *X. bromi*, *X. campestris*, *X. cassavae*, *X. codiaei*, *X. cucurbitae*, *X. fragariae*, *X. hortorum*, *X. melonis*, *X. oryzae*, *X. pisi*, *X. populi*, *X. vasicola*, and *X. vesicatoria* (Rodriguez et al., 2012). Thus, taxonomy of this genus are still subjected to debate since the last decade (Rodriguez et al., 2012; Vandroemme et al., 2013; Lamichhane, 2014).

## Conclusion

The taxonomy of all these macergens is far from being complete because of the controversial issues arising from their classification which were based on host pathogenicity (Table 1). This may be affected by the sudden change in the ecosystem. This classification is not based on scientific research perspective for defined taxa and the consequences brought about by these marcergens may become difficult to understand. It is majorly based on symptoms that is similar in all the macergens, and this is unreliable according to (Sławiak et al., 2013). Although, some scientific method like MLSA were used for the classification they have limitation of single locus analysis. Thus, a proper classification is imperative, in order to reflect an understanding of their existing natural diversity and relationships among them. This will help plant breeders, farmers, and legislators to ensure quick and effective disease diagnosis and management, in order to avoid unnecessary destruction of economically valuable crops. The knowledge of genomic diversity within the macergens pathovars is necessary for host resistance disease based management strategies for the plant breeders.

As a concluding comment, we would like to stress that we applaud further developments in molecular methods of analyzing macergens for a better classification of these macergens. It is our belief, however, that any future progress in taxonomy as a scientific discipline will depend only on the availability of new experimental data that will broaden and refined the view on bacterial diversity.

## Author Contributions

BR involved in data collection from internet, drafting of the manuscript or revising it critically for important intellectual content; have given final approval of the version to be published; and agree to be accountable for all aspects of the work in ensuring that questions related to the accuracy or integrity of any part of the work are appropriately investigated and resolved. OO involved in collection of data, drafting of the manuscript, revising it critically, responsible for any aspect of the article and also help in the general supervision of the article.

### Conflict of Interest Statement

The authors declare that the research was conducted in the absence of any commercial or financial relationships that could be construed as a potential conflict of interest.

## Abbreviation

DNA: Deoxy Ribonucleic Acid
ITS: Internal transcribed spacer
MLSA: Multilocus Sequence Analysis
NADH: Nicotinamide Adenine Dinucleotide (Reduced)
RNA: Ribonucleic Acid
rDNA: Ribosomal Deoxy Ribonucleic Acid
rRNA: Ribosomal Ribonucleic Acid
